# *Peganum harmala* Aqueous and Ethanol Extracts Effects on Lesions Caused by *Leishmania major* (MRHO/IR/75/ER) in BALB/c Mice

**DOI:** 10.5812/jjm.10992

**Published:** 2014-07-01

**Authors:** Fariba Khoshzaban, Fatemeh Ghaffarifar, Hamid Reza Jamshidi Koohsari

**Affiliations:** 1 Department of Parasitology and Mycology, Faculty of Medical Sciences, Shahed University, Tehran, IR Iran; 2 Department of Parasitology, Faculty of Medical Sciences, Tarbiat Modares University, Tehran, IR Iran

**Keywords:** Cutaneous Leishmaniasis, Treatment, Peganum harmala, Ethanol

## Abstract

**Background:**

Leishmaniasis is one of the six most common parasitic infections in the tropical regions. There are different therapeutic modalities, however therapeutic resistance is developed and resulted in numerous problems. Therefore, evaluation of other therapeutic modalities is performed extensively.

**Objectives:**

The current study aimed to compare the therapeutic response of cutaneous leishmaniasis with Glucantime and *Peganum harmala* extracts (aqueous and ethanol) in the animal model.

**Materials and Methods:**

The therapeutic response of *Leishmania major* to Glucantime and *P. harmala* extracts (aqueous and ethanol) in animal model was studied in BALB/c mice. These mice were divided into four groups according to receiving either one of these three agents, and the control group. The therapeutic response was evaluated according to the parasitic load before and after treatment and also with measuring the size of the lesions.

**Results:**

The results showed that ethanol extract of *P. harmala* had good therapeutic efficacy in treatment of lesions in mice (P < 0.05), and the efficacy was significant in the eighth week after the treatment. There was also a statistically significant difference between the groups regarding the parasitic load (P < 0.05).

**Conclusions:**

According to the current study results, it may be concluded that ethanol extract of *P. harmala* is efficient in the treatment of cutaneous leishmaniasis, and the efficiency is comparable with that of Glucantime.

## 1. Background

Cutaneous leishmaniasis is often self-healing, particularly in infection with *Leishmania major*; therefore, medical treatment is not always recommended, however, if lesions do not spontaneously heal, within six months or when the lesions are especially disfiguring in a cosmetically sensitive area, treatment is required. Even though lesions may eventually heal in the absence of treatment, the process is often long and produces significant scare; thereby it justifies the use of chemotherapy. The goal of treating cutaneous leishmaniasis is two-fold, eradication of amastigotes as well as reducing the size of the lesions; so that healing will take place with minimal scare (1, 2). *Leishmania* spp. can escape from the killing system of macrophages, stay alive inside them, and propagate (1). 

Various methods have been presented for medical treatments of the disease. The most effective drug is the pentavalent antimony. But it is expensive, rare, has fairly severe side effects, and to be effective it requires long term of application. Moreover, the effects of various formulations are different and owing to drug resistance, its efficiency has decreased in recent years (3, 4). The use of natural products in the treatment of a variety of diseases has increased due to the considerable number of medicinal plants with proven biological activity applicable to the treatment of some diseases (5). Over the years, the World Health Organization (WHO) has advocated traditional medicines as safe remedies for both microbial and non-microbial diseases (6).

One of the most famous plants used in the popular medicine is *Peganum harmala*. It is a perennial herbaceous glabrous plant and grows in semi-arid rangeland and sandy soils, especially along the Mediterranean regions in North Africa and the Middle East (6). *P. harmala* L. (Zygophyllaceae), the so-called ‘‘Harmal’’or “Espand” is native in the steppe areas of semiarid and pre-desert regions, such as Iran (7). Several reports have been published in the literature on the antimicrobial activity of alkaloids of *P. harmala* (8). Apart from the widespread use of these b-carboline alkaloids, which show monoamine oxidase inhibition and are used as a psychoactive drug to treat Parkinson, Astulla et al., Di Giorgio et al., and Rahimi-Moghaddam et al. have exhibited various bioactivities such as, cytotoxicity against human cancer cell lines, anti-bacterial, antitumoral, and anti-oxidant activities, enzyme inhibition, immunomodulator properties, vasodilator activity on rat aorta, and anti-leishmanial activity toward parasites of *L. infantum* (9-11).

## 2. Objectives

The current study, after developing the cutaneous leishmaniasis lesion in susceptible animal models, aimed to treat the animals with currently available drugs (pentavalent antimony) as well as the aqueous and ethanol extract of *P. harmala* and then analyze the results with respect to the size of lesion, duration of treatment, presence of the parasite in the site, remaining of scar, and mortality of the mice.

## 3. Materials and Methods

### 3.1. Preparation of P. harmala Extracts and Ointment

#### 3.1.1. Preparation of Aqueous Extract of P. harmala

The required amount of the ground seed of the plant (1000 g) was obtained, 400 mL of distilled water was added, and then the solution was slowly heated and simultaneously stirred until the early signs of boiling emerged. At that time, the solution was taken off the heat and filtered using Wattman No. 42 filter paper. The filtered solution was taken to the ban marry bath at 90 to 100°C, until the extract got a gel form. The gel was ready for weighing and solving in appropriate solvent (sterile distilled water). After preparing the solution, the extract was filtered using sterile needle filter 45% (12).

#### 3.1.2. Preparation of P. harmala Ethanol Extract

The required semi-grinded amount of the plant seed (1000 g) was added to 400 mL of ethanol in room temperature and light, and then the solution was stored for two to three days. In these days, the solution was occasionally shaken for five minutes and then filtered by Wattman No. 42 filter paper. The filtered solution was taken to the ban marry bath at 60 to 65°C, until the ethanol portion was evaporated and a gel form material was obtained. Then, 500 g of the gel was solved in ethanol solvent and the obtained solution was filtered using sterile needle filter 45% (12).

#### 3.1.3. Preparation and Administration of Ointment of Aqueous and Ethanol Extract of P. harmala

After weighing and determining the required amount of effective material, the ointment was prepared on the basis of essence-free Vaseline according to IC50, which was 40 and 35 μg/mL for water and ethanol solutions, respectively. The ointment was used once daily for eight weeks.

### 3.1.4. The Experiment Steps and Treatment

The mice were evaluated with regard to the progression or improvement of the disease (duration of the lesion, size of the lesion during treatment, healing, presence of the parasite at the site of lesion, and mortality).

### 3.2. Animal Model of Leishmaniasis Lesion

Forty male BALB/c mice aged between seven and eight weeks with 20 to 25 g weight were purchased from Pasteur Institute of Iran.

### 3.3. Development of Leishmaniasis Lesion in the Mice

The parasites used in the experiment were of the standard strain of leishmaniasis in Iran (MRHO/IR/75/ER), stored in BALB/c strains mice. After isolation of the parasites from the mice, the parasites were propagated in bi-phasic Novy-MacNeal-Nicolle medium (NNN) and single-phasic RPMI-1640 containing 10% FCS (Fetal Calf Serum) media, and then with the aim of developing leishmaniasis lesions, the mice were inoculated with the parasites. After obtaining sufficient parasites in NNN and RPMI media to contaminate the mice, the parasites of the third passage on the RPMI media whose severity had not decreased were used at maximum. Before injection, the tail of mice was shaved and then 100 µL of the parasites (107/mL) in the static phase was injected per mice (11).

### 3.4. Categorization of Mice

The mice with lesion were evaluated for leishmaniasis lesion with microscopic test and observation of the Leishman body. Then, they were classified into four groups. One group was considered as the control group and did not receive any treatment to evaluate the progression of the disease, duration of lesion, size of the lesion during the course of the disease, spontaneous healing, mortality of the animals, and presence of the parasite in liver and spleen of the animals. The three other groups were treated.

### 3.5. Treatment

#### 3.5.1. Treatment With the Pentavalent Antimony (Glucantime)

According to the WHO protocol, the drug was injected into the lesion site and in different points by insulin syringes (12). Treatment with aqueous and ethanol extract of *P. harmala* which was formulated as ointment (12).

### 3.6. Liver, Spleen, and the Lesion Sampling at the End of the Experiment (End of the Eighth Week)

At the end of the eighth week, samples were taken from the lesions. The liver and spleen samples were provided after scarifying the animals to evaluate the capability of the parasite in proliferation and involvement of other organs. The samples were dried out at the laboratory temperature, fixed with methanol 100%, and then dyed with Geimsa stain. The prepared slides were microscopically evaluated (×100). The presence of the parasite in different organs was ranked according to the following definition: The number of parasites in different microscopic fields was counted for each 100 tissue cell nuclei. Presence of Leishman body was considered a positive finding and if no parasite could be observed through the slide, it was considered negative. Two liver and two spleen samples were taken from each mouse.

### 3.7. Determining the Parasite Load of the Lesions and Survival Rate

Samples were taken from the lesions at the beginning (before applying the ointment containing aqueous and ethanol extracts of *P. harmala* and injection of Glucantime) and also at the end of the treatment (before scarifying the mice), and the contamination severity (parasite load) was determined according to the following definition:

If in ten microscopic fields with oil lens (×100), an average of 1, 1-10, 11-100, and 101-1000 Leishman bodies were observed, they were scored as +1, +2, +3, and +4, respectively, and if no parasite could be observed through the slide, it was considered negative. The results were recorded in a table and then analyzed. The survival rate for all groups was checked weekly. The collected data were analyzed using SPSS software version 15, employing chi-square, ANOVA, Tukey, and measure tests, at the significance level P < 0.05. 

## 4. Results

After developing the lesions and before treating the mice, lesion samples were taken to evaluate the parasite infection (Table 1). The highest parasite load observed in different groups was as follows: +2 for the Glucantime group, +2 for the ethanol extract of *P. harmala*, + 3 for the aqueous extract of *P. harmala*, and + 2 for the control group.

**Table 1.** Parasite Load of Primary Lesion Samples (Before Treatment)^a^

Group	+1	+2	+3	+4
**Glucantime**	30	50	20	0
**Ethanol extract of ** ***P.harmala***	0	70	30	0
**Aqueous extract of ** ***P.harmala***	0	30	60	10
**Control**	0	60	30	10

^a^Data are presented in %

At the end of the treatment, lesion samples were prepared again. The microscopic results are provided in Table 2. As indicated, after treatment, 40% of mice in the Glucantime and the ethanol extract groups become parasite-free, while in the aqueous extract group only 10% of mice healed. As expected, negative cases were not observed in the control group.

**Table 2.** Parasite Load of the Secondary Lesion Samples (After Treatment)^a^

Group	Negative	+1	+2	+3	+4
**Glucantime**	40	40	10	10	0
**Ethanol extract of ** ***P.harmala***	40	30	20	10	0
**Aqueous extract of ** ***P.harmala***	10	60	30	0	0
**Control**	0	0	40	40	20

^a^Data are presented in%

By comparing the parasite load before and after treatment in the Glucantime group (Table 3), it can be observed that at the end of the treatment 40% of the mice were parasite-free and the maximum parasite load in other mice changed from + 2 to +1. In general, a significant difference was observed before and after treatment in the Glucantime group with respect to parasite load (P < 0.05).

**Table 3.** Parasite Load Before and After Treatment in the Glucantime Group^a^

Group	Negative	+1	+2	+3	+4
**Before treatment**	0	30	50	20	0
**After treatment**	40	40	10	10	0

^a^Data are presented in%

Comparing the parasite load before and after treatment in the ethanol extract group (Table 4) showed that at the end of the treatment, 40% of the mice were parasite-free and the maximum parasite load changed from +2 to +1. In general, a significant difference was observed before and after treatment in the ethanol extract group with regard to parasite load (P < 0.05).

**Table 4.** Parasite Load Before and After Treatment in the Ethanol Extract Group ^a^

Group	Negative	+1	+2	+3	+4
**Before treatment**	0	0	70	30	0
**After treatment**	40	30	20	10	0

^a^Data are presented in%

By comparing the parasite load before and after treatment in the aqueous extract group (Table 5), it is obvious that at the end of the treatment 10% of the mice were parasite-free and the maximum parasite load in the other mice changed from +3 to +1. With respect to parasite load, a significant difference was observed before and after treatment in the aqueous extract group (P < 0.05).

**Table 5.** Parasite Load Before and After Treatment in the Aqueous Extract Group^a^

Group	Negative	1 +	2 +	3 +	4 +
**Before treatment**	0	0	30	60	10
**After treatment**	10	60	30	0	0

^a^Data are presented in%

Considering the changes in the size of lesions during the eight weeks of the treatment, it can be observed that in all the groups under study, the size of lesions decreased up to week six and then in weeks seven and eight, the lesion size did not change in the Glucantime and aqueous extract groups while it decreased in the ethanol extract group in the last two weeks (Table 6). In the control group, as expected, during the study the lesion size increased continuously. The results of measure test showed that in different weeks, the size of lesions decreased significantly in the Glucantime, ethanol, and aqueous extract groups, while such a difference was not observed in the control group.

The sizes of the lesions were evaluated at the end of the experiment using ANOVA and it was found that the groups were significantly different in this respect (P < 0.05). The results of Tukey test demonstrated that the observed difference resulted from the difference in the effect of Glucantime, ethanol, and aqueous extracts. However, the Glucantime and ethanol extract groups were not significantly different with respect to the size of the lesions. In other words, it can be concluded that Glucantime and ethanol extracts have the same effect on reduction of the lesion size and their effects were greater than those of the aqueous extract and control groups. At the end of the experiment liver and spleen sampling was carried out to evaluate the reproduction of the parasite and its ability to involve other organs. In the prepared liver specimens, 80%, 70%, and 30% of the Glucantime, ethanol and aqueous extract groups were parasite-free, respectively.

**Figure 1.**
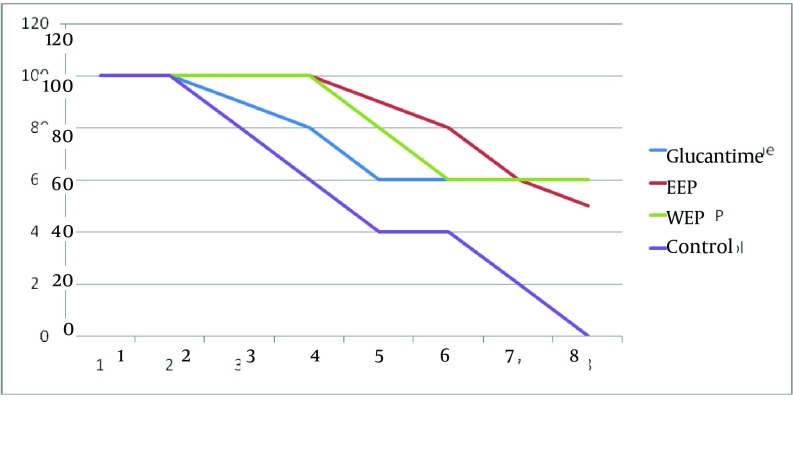
The Survival Rate of the Treatment and Control Groups During eight Weeks EEP, Ethanol Extract *P. harmala*; WEP, Aqueous Extract *P. harmala*

The results of the spleen specimens were as follows: 60%, 70%, and 20% of the Glucantime, ethanol, and aqueous extract groups were parasite-free, respectively. It should be noted that all the liver and spleen specimens of the control group were contaminated with the parasite. At the end of the treatment, in each of the Glucantime, ethanol, and aqueous extract groups, six mice survived, while in the control group all mice died by the end of the week seven. In the Glucantime group, three cases of treatment were observed, which occurred in weeks four, five, and six. Four cases of treatment were observed in weeks three, five, six, and seven in the ethanol extract group and one case of treatment in week six in the aqueous extract group (Figure 1).

**Table 6.** Frequency Distribution ± SD of the Mean Size of Lesions in Different Groups During the Course of Treatment

Week	Glucantime	Ethanol Extract	Aqueous Extract	Control
**Before treatment**	11.85 ± 1.87	9.95 ± 3.10	11.10 ± 4.6	10.40 ± 2.40
**First week**	11.30 ± 3.39	9.31 ± 2.31	9.70 ± 5.03	10.75 ± 2.32
**Second week**	9.90 ± 4.49	9.90 ± 4.02	8.90 ± 4.71	11.40 ± 2.29
**Third week**	7.66 ± 2.26	9.85 ± 4.22	8.80 ± 4.38	12.21 ± 1.70
**Fourth week**	6.55 ± 2.51	8.75 ± 4.60	7.80 ± 3.48	12.75 ± 1.29
**Fifth week**	5.27 ± 1.54	8.45 ± 5.17	6.75 ± 2.87	12.87 ± 1.93
**Sixth week**	4.64 ± 1.40	6.27 ± 4.62	5.58 ± 1.42	13.62 ±2.09
**Seventh week**	4.10 ± 1.46	3.42 ± 3.33	5.00 ± 1.37	15.75 ± 1.06
**Eighth week**	3.83 ± 1.16	1.83 ± 0.93	4.91 ± 1.59	All mice died

## 5. Discussion

Leishmaniasis is a parasite which causes a wide range of diseases and is more or less reported around the world (13). The history of using herbal drugs to treat diseases goes back to the early years of mankind history. Later during the centuries, the use has been developed (14). Natural forms of the plants or their extracts are widely used against pathogenic microorganisms (7). Considering the particular climate and geographical condition of Iran, many medicinal plants can easily grow in Iran and the country is considered as one of the most appropriate places for growing such plants throughout the world (5). Treatment of cutaneous leishmaniasis should lead to eradication of the parasite and elimination of the amastigote from the lesion, which would finally lead to reducing the size of the lesion and its healing (14). However, until now no medical treatment can perfectly meet these criteria. 

Employment of medicinal plants has been common for years; for instance, amaranth has been used as an anti-malaria drug (15). Alkaloids are among the most important natural extracts used by man (16). The effect of alkaloids has been evaluated against Giardia spp. and *Entamoeba histolytica* in different studies (17). The effects of Berberine, as a major group of alkaloids, against *Trypanosoma* spp. and plasmodium spp. is approved (18). *P. harmala* is commonly used as a disinfectant and its oral use is contraindicated owing to its toxicity (19). The seeds of *P. harmala* contain different alkaloids such as Harmin, Harmalin (harmidine and harmalol methyl ether), as well as Harmalol (7).

In patients with leishmaniasis, treatment of cutaneous lesions is one of the main objectives. To achieve this goal, treatments that are not systemic and have fewer side effects are more desirable. Thus, the current study compared the therapeutic effect of water and ethanol extracts of *P. harmala* with that of the chemical drug Glucantime, as one of the common drugs of the disease. Is spite of being toxic, alkaloids have been used in Iranian traditional medicine, and many alkaloids have been used in treatment of parasitic diseases. *P. harmala* is a plant that contains alkaloids with IC50 40 μg/mL. 

A study demonstrated that the number of amastigotes in macrophages decreased from 1.5 to 0.7 after 60 hours of exposure to 40 μg/mL of *P. harmala* extract, while the number reached 2.3 in the control group (20). Considering the results obtained by Yousefi et al. (20), the current study considered the concentration of 40 μg/mL in the ointment. As indicated in the results, the highest efficacy was observed in the samples obtained from the groups treated by Glucantime and ethanol extract of *P. harmala*. However, parasite load reduction was also observed in the group that received aqueous extract of *P. harmala*. The findings indicated that ethanol extract of *P. harmala* has similar effects to those of the routine chemical treatment of the parasite, Glucantime. Mirzaie et al. found that *P. harmala* extract inhibits the growth of promastigotes of *L. major* in 72 hours on media (19).

Lala et al. evaluated the anti-leishmaniasis characteristics of Harmin, which is a beta-carbolin alkaloid amine extracted from *P. harmala* seeds, in different vesicular release systems (14). In the current study two liver samples as well as two spleen samples were prepared from each mouse in the groups and were microscopically evaluated. In the Glucantime, ethanol, and aqueous extract groups, 60%, 70%, and 30% reduction in the parasite contamination was observed, respectively; while in all specimens taken from the control group, the parasite was observed. With respect to the ability to remove visceral elimination of the parasite, the results indicated that ethanol extract has similar effects to that of the Glucantime group. In the study carried out by Tabatabaie et al. the in-vivo effect of *P. harmala* on *L. major* was evaluated. Their results showed similar effects with the application of *P. harmala* seed ointment and Glucantime (12). This is consistent with the findings of the current study. Tabatabaie et al. investigated the oral administration of *Ferula asafoetida *and *P. harmala* in the treatment of *L. major *infection. It was demonstrated that the best therapeutic effect can be achieved by simultaneous application of Glucantime and herbal ointment and the oral drug (12). In this study, after eight weeks of treatment, the lesion size reduced in the groups under treatment, and the reduction was similar in the Glucantime and ethanol extract groups. Based on the observations in the above-mentioned and the current studies, and since the extracts (aqueous and ethanol) of *P. harmala* seeds were used and the observed therapeutic effects were similar to that of the chemical drugs, it can be concluded that the current study obtained better results.

According to the current study results, employment of herbal extracts can be considered in the treatment of patients with leishmaniasis. Moreover, regarding the characteristics of *P. harmala* and the therapeutic effects of the plant extracts, particularly its ethanol extract, can be considered as an appropriate treatment choice in patients with leishmaniasis. However, further research is required to determine the minimum therapeutic dose of the drug for the ethanol extract, as well as its intra-lesion injection similar to the Glucantime application, and the possibility of testing the drug on human cases. Such studies would reveal the scientific and practical potentials of this medicinal plant.
